# Exploring the relationship between housing conditions and Métis Nation of Ontario citizen’s health: a qualitative study

**DOI:** 10.1186/s12889-025-23716-x

**Published:** 2025-07-25

**Authors:** Noel Tsui, Abigail J. Simms, Carolyn Lacka, Helana Marie Boutros, Robynn Sadler, Amy Mersereau, David Crenna, Jeff Evenson, Cindi Rye, Sarah A. Edwards

**Affiliations:** 1 Métis Nation of Ontario, 66 Slater Street, Ottawa, ON K1P 5H1 Canada; 2 ICES Central, V1 06, 2075 Bayview Avenue, Toronto, ON M4N 3M5 Canada; 3 Dalla Lana School of Public Health, University of Toronto, 155 College St, Toronto, ON M5T 3M7 Canada; 4 Department of Health, Aging and Society, McMaster University, 1280 Main Street West, Hamilton, ON L8S 4L8 Canada; 5 Priority Decision Data, Ottawa, ON K1Y 1H1 Canada; 6 Edgewood Associates, Toronto, ON M4W 3A9 Canada

**Keywords:** Métis, Indigenous health, Social determinants of health, Housing, Métis determinants of health

## Abstract

**Background:**

Housing is an important social determinant of health as the lack of housing or substandard housing conditions can negatively impact people’s health and wellbeing. Indigenous Peoples in Canada are three times more likely to live in substandard housing than non-Indigenous people. The objective of this study is to examine the thoughts, feelings, and stories of citizens of the Métis Nation of Ontario (MNO) on how their housing conditions impact their health.

**Methods:**

Thirty-five (35) MNO citizens were recruited for the study, and seven focus groups were conducted between August 2022 and February 2023. All focus groups were conducted via Zoom with 3–9 participants, one facilitator, one note-taker, and one MNO Senator. All focus groups were recorded. Each transcript was coded and analyzed using thematic analysis by two independent coders.

**Results:**

Seven themes were derived from all focus groups: housing needs and conditions, affordability of shelter costs, renting, infrastructure and connectedness, impacts of housing on mental and emotional health, impacts of housing on physical health, and improvements to housing programs and supports.

**Conclusions:**

This is the first Métis-specific study to explore and gather the lived experiences and stories of MNO citizens regarding the impact of housing conditions on their health. Our findings revealed a multifaceted relationship between housing and health, extending beyond individual’s living conditions, with a significant impact on Métis citizens’ mental health. The results will be used to inform MNO housing programs to increase Métis homeownership and improve affordable housing options.

## Background

Housing is often cited as an important social determinant of health, as it acknowledges the effects of inadequate housing on peoples’ health and wellbeing [1–3]. In consultation with Métis citizens living across Canada, following definition of health for Métis people has been suggested:

*“Health is a state of complete physical*,* mental and social wellbeing and not merely the absence of disease or infirmity”. It is a state of balance and interconnected relationships between physical*,* mental*,* emotional*,* social*,* financial/economic*,* spiritual*,* environmental*,* and cultural wellbeing. And it is the extent to which Métis people*,* families*,* or communities can achieve individual or collective wellbeing now and for future generations”* [3].

Housing is also recognized as one of the Métis social determinants of health [3].

Poor housing conditions have been shown to be associated with lower self-reported health status and more doctor visits [4], poorer mental health [5], and various health issues, such as respiratory infections, asthma, and lead poisoning [2]. Poor housing conditions have also been found to have a negative effect on one’s cultural and spiritual wellbeing [6]. Living in neighbourhood with nearby green space is associated with better health outcomes, including reduced risk of mortality, cardiovascular disease, and better psychological health [7]. Limited access to green space, particularly in urban areas, can be especially impactful for certain populations, like Indigenous Peoples, whose cultures are inherently connected to the land [7, 8]. Exposure to substandard housing is also not experienced equally among different populations [2, 9]. Individuals who have low-income status and marginalized populations, like Indigenous Peoples in Canada, carry a greater risk of disproportionately experiencing poor housing conditions [10].

In the Canadian context, adequate housing refers to a dwelling not in need of major repairs, such as defective plumbing or electrical systems, or necessitating structural renovations to walls, floors, or ceiling [11]. Suitability is also tracked, defined as enough bedrooms for the size and composition of the household, according to the National Occupancy Standard (NOS) [12]. Affordability is also monitored, defined as spending less than 30% of before tax income on housing [12]. If one or more of these three measures are not met and a household would have to spend 30% or more of before tax income to move to suitable alternative housing, a household is considered in core housing need [12]. According to the 2021 Canada Census, 13.6% of Indigenous Peoples live in core housing need [13]. Over one in six Indigenous People lived in unsuitable housing, 16.4% lived in a dwelling that required major repairs, and 16.8% were living in unaffordable housing [14]. Specifically, 9.7% of self-identified Métis people were in core housing need with 15.7% living in unaffordable housing, 10% were living in a dwelling in need of major repairs, and 7.9% were living in unsuitable housing, particularly in census metropolitan areas [15]. While these findings describe self-identified Métis housing experiences, they may not accurately represent Métis People who are registered citizens and rights holders within of the Métis Nation. For example, when citizens of the Métis Nation of Ontario (MNO) were asked about the state of their homes in a household survey led by the MNO, 23% of MNO citizen’s homes required major repairs [16].

The Métis are one of the three Indigenous Peoples constitutionally recognized in Canada, with their own unique culture, language, and way of life [17, 18]. They originated primarily from the unions between First Nations women and European men, which led to the ethnogenesis of a distinct people who emerged in the historic Northwest, including the Upper Great Lakes and waterways of northern Ontario in the late 1700 s [19].The MNO is currently the only federally and provincially recognized Métis government in Ontario, Canada’s most populous province, and represents the collective aspirations, rights, and interests of Métis citizens throughout the province [20, 21]. The MNO is governed by the Provisional Council of the Métis Nation of Ontario (PCMNO). Province-wide ballot box elections are held every four years where MNO citizens elect representatives of 9 regions in Ontario, in addition to the President, Chair, Vice-Chair, Secretary-Treasurer and Executive Senator. The MNO is one of several Métis governments throughout Canada including the Manitoba Métis Federation, Métis Nation-Saskatchewan, Otipemisiwak Métis Government (formerly Métis Nation of Alberta), and Métis Nation British Columbia.

Currently in Ontario, there is a need to build one million new homes [22]. If an average household were to purchase an average home in Ontario in 2021, they would have to spend 60% of their income on housing costs [22], which is double the recommended income-to-housing ratio for affordable housing [11]. Given the urgency to address the continuously growing housing crisis in Ontario, the MNO provides housing programs, including first-time home-buyer support, emergency repairs, and building affordable housing units, to support their citizens through this critical time. Some Regional Councils of the MNO have also endeavored to create their own local affordable housing complexes [23]. The objective of this study is to examine MNO citizens’ thoughts, feelings, and stories on how their housing conditions have impacted their health. This study will alleviate the current paucity of Métis-specific health research and will inform MNO housing programs with the goal of increasing Métis homeownership and improve affordable housing options.

## Methods

### Study design and setting

This study used convenience sampling to recruit participants through existing MNO social media channels and the MNO website. MNO citizens interested in participating were directed to an online screening survey using Qualtrics survey software. This survey first obtained participants’ informed consent, then collected personal information (for citizenship verification and focus group scheduling purposes), current housing situation, and basic demographic information (e.g., MNO region of residence, age, and gender). Eligibility criteria included being (i) a registered MNO citizen, (ii) a current resident of Ontario, and (iii) 18 years of age or older. This study received ethical approval from the University of Toronto Health Sciences Research Ethics Board (REB#42320).

Recruitment and focus groups were conducted between August 2022 and February 2023. The structured focus group interview guide (questions are provided in Table 1) and survey were co-developed by MNO staff members, leadership, and citizens, as well as housing and research experts.

**Table 1:** Focus group interview guide. The probing questions are not included

Focus Group Guide Questions	
1.	Please describe what is a healthy home from your perspective and what contributes to or is important for healthy and good housing?
2.	In what ways can the quality of a person’s housing affect their health and wellbeing?
3.	Please tell us about the current condition of your home?
4.	Do you think the condition of your housing (current or previous) has had, or had an impact on your overall health (i.e., physical, mental, emotional, or spiritual)?
5.	What are the most pressing housing needs in your region?
6.	What is your housing story?
7.	How has the COVID-19 pandemic affected your housing situation?
8.	In your view, which (group of) MNO citizens are most in need of MNO housing program services?
9.	How can the MNO ensure that every Métis citizen and household has an affordable, comfortable, safe and secure place to live? What would you put that effort in the list of MNO’s priorities?
10.	Of all the points brought up in our discussion, what is the single most effective thing the MNO can do to address the housing needs of MNO citizens and to help ensure citizens stay healthy and well?
11.	Is there anything we did not discuss that should have been discussed?

Upon consent to participate, MNO citizens were contacted by a research team member (NT) to schedule the focus group dates and time. Each focus group comprised of 3–9 participants, one facilitator (NT or AJS), one note-taker (NT or AJS), and one MNO Senator. All focus groups were conducted online via Zoom and were recorded with participants’ consent. All participants received the question list and Zoom instruction guide via email prior to the focus group to allow for preparation. A thank you email containing MNO mental health resources and a link to an additional survey on the acceptability of virtual focus groups was emailed to participants after the focus group. A summary of the focus group discussion was emailed to each participant for participant verification once transcripts were downloaded from Zoom and anonymized.

All participants received a $50 grocery store gift card upon completion of the focus group and an additional $5 coffee gift card if they completed a voluntary survey about their experience with virtual focus groups.

### Analysis

Transcripts were coded and analyzed using thematic analysis to inductively develop themes. Three coders (NT, CL, HMB) finalized the codebook per each transcript and included comprehensive codes that reflected the definitions of the themes and subthemes finalized. The codebook was reviewed by AJS for additional input and clarity. Two coders (NT plus CL or HMB) independently coded each transcript. Any discrepancies were thoroughly discussed between coders and consensus was achieved. The codebook is presented in Table 2.

**Table 2:** A summary of the themes, subthemes, and definitions used in our analysis

Themes	Sub-themes	Definitions
Housing needs and conditions	Major repair	A repair that is necessary to ensure the continued habitability of the house or extend the useful life of an asset (e.g., furnace, safety/accessibility upgrades, windows, roof, plumbing, etc.)
	Minor repair	Low-cost repairs to existing equipment or cosmetic repairs (e.g., replacing door locks, light bulbs, leaky faucets, etc.)
	Lack of space	Insufficient, shortage, or absence of space required or desired
	Mold or mildew	The presence of mold or mildew inside housing
	Overcrowding	The presence of more people in a space than is comfortable, safe, or permissible
	Pest	Any animal or insect harmful to humans or human concerns
Renting	Landlord-tenant relationship	Experience with landlords
	Long wait list for housing programs or assistance	Long wait time for housing programs or assistance
Affordability of shelter costs	Rent/mortgage payments	Rent or housing price or mortgage amount is too much for people to afford with the current cost of living or economy
	Buying a home	Unable to purchase a home due to high prices
	Cost of housing repairs	The cost of repairs or maintenance is too high or unaffordable
	Inflation and supply chain delays	Inflation and supply chain delays caused by the COVID-19 pandemic
	Housing shortages	A deficiency or lack in the number of houses needed to accommodate the population of an area
	Housing insecurity	The lack of security or stability in an individual shelter
	Poor housing quality	Housing that has inadequate conditions
Infrastructure and connectedness	Community safety	Participants feeling unsafe in housing (e.g., high crime rate in neighbourhood, discrimination, etc.)
	Lack of contractors	Difficulty or inability to find contractors in a particular region due to various factors
	Environmental contamination	Environmental contamination near current or previous housing
	Lack of greenspace	Lack of greenspace around housing
	Distance from amenities	Distance from amenities such as public transportation, bank, hospital, school, etc.
	Access to community/family	Experience of having or lacking access to community and family due to location of housing
Impacts of housing on Mental and Emotional Health	Feeling stuck	Participant expressed feeling stuck at their current or past housing (e.g., due to financial issues, unable to find another place, must stay close to work, etc.)
	Stress/anxiety	Stress or worry about everything housing related
	Social pressure	Social pressure about their housing situation, size of their home, cleanliness, etc.
	Housing supporting mental health	Better housing quality resulting in better mental health outcomes
	COVID-19	Direct or indirect impact of housing on mental health caused by the COVID-19 pandemic
	COVID-19 stay at home mandate	Experience with the COVID-19 stay at home mandate
Impact of housing on Physical Health	Respiratory illnesses	Respiratory illnesses such as asthma, allergy, coughing caused by housing
	Other illnesses	Other illnesses/impact on physical health caused by housing related issues
Improvements to housing programs and supports	MNO-Owned housing	Participants’ suggestions for Métis community housing or MNO-owned housing
	Expand existing programs and develop new program	Participants’ suggestion to develop new program/support (e.g., housing legal advice for tenants, snow removal assistance) and expand existing programs
	Reduce program barriers	Participants’ suggestion to reduce program barriers (e.g., simplify the application process, provide clear instructions, streamline resources across regions, etc.)
	Re-evaluate eligibility criteria	Participants’ suggestion to re-evaluate the eligibility criteria and income threshold for housing assistance/program to better reflect the current economy
	Better advertise programs and support	Participants’ suggestion to better advertise existing housing programs (e.g., MNO, federal, provincial, or other Indigenous organizations)

Hennink et al. [24] indicates that code and meaning saturation can often be achieved after four to five focus group discussions. However, the concept of saturation is linked to specific non-Indigenous methodologies. Our study considers information power, which is influenced by the study’s aim, the specificity of the sample, high quality dialogue, and our unique analysis strategy [25]. Therefore, our research adopts an Indigenous reflexive approach that rejects the neo-positivist approach of measuring data redundancy, favouring a generative process of interpretation through thematic analysis [25, 26]. This approach emphasizes finding meaning in how things relate to each other and supports the creation of shared narratives [27]. It involves researchers reflecting on their positionality and perception of the participants’ experiences during data collection and/or analysis, sharing these reflections with participants, and allowing participants or community to be recognized in the process [27]. Participant verification gives participants the opportunity to review the data generated from these focus group conversions that will be used in the analysis and further involves them in the analytical process. This can help strengthen the researcher-participant relationship. For this study, 39 MNO citizens completed the screening survey and consented to participate. In total, 35 participants completed the study, and 7 focus groups were conducted. Four MNO citizens who completed the screening survey did not participate in the focus groups due to not joining the Zoom call or lack of response to invitations to participate in a focus group.

## Results

### Demographics

Demographics of participants are shown in Table 3. Counts less than 5 are suppressed in Table 3 to minimize the risk of re-identification of participants. Most (83%) of the participants identified as women. Half (53%) of the participants were age 45 and above. A majority (69%) of the participants currently live in their own home or a home owned by other family members, and the remaining participants were renting.

**Table 3:** Demographics of participants/mno citizens participated in focus groups from August 2022 to February 2023

Demographic		*N* = 35
Gender		
Woman		30 (83%)
Man		< 5
Non-Binary or Two-Spirit or other gender		< 5
Age		
18–24		< 5
25–44		16 (44%)
45–65		15 (42%)
65+		< 5
Region		
1–4		11 (31%)
5–7		16 (46%)
8–9		8 (23%)
Current housing		
Rent		10 (31%)
Own		25 (69%)

### Thematic analysis

Our analysis of the conversation had during each focus groups identified 7 themes: housing needs and conditions, renting, affordability of shelter costs, infrastructure and connectedness, impacts of housing on mental and emotional health, impacts of housing on physical health, and improvements to housing programs and supports.

Across all 7 focus groups, participants shared the need for both major and minor repairs. In most of the focus group discussions, participants spoke about unaffordable housing, housing shortages, lack of space, the presence of mold and mildew in their homes, discrimination from landlords, and unsafe living environments. These issues were not experienced in isolation, participants often described how multiple housing challenges overlapped and compounded, leading to impacts on their mental and physical health, such as feeling stress and worried and developed respiratory illnesses. These themes, along with their sub-themes were further explored below.

#### Housing needs and conditions

This section explores the impact that aspects of the physical dwelling, such as needed major and minor repairs, overcrowding, pests and other environmental concerns, have on the wholistic wellbeing of our focus group participants.

##### Need for repairs

Most participants described the need for both major and minor repairs in their homes, often linking these issues to feelings of stress, helplessness, and declining wellbeing. These unmet repair needs affected their daily lives, contributing to mental strain and a sense of instability. Mental strain included feeling stressed and helpless about their housing needing major or minor repairs that they were not able to address. As a participant from focus group 6 describes:

*“My floor is sinking and I can see it. I can feel it. It’s very drastic*,* and I see it every day*,* and it’s like a constant reminder. It’s like*,* okay*,* I have this. It needs to be fixed*,* but I don’t have the finances by myself.”* (FG#6)

The inability to address a major structural issue with their housing weighed on them. Overall, participants described living in housing that needed major repairs such as structural issues integrity (e.g., damaged roof, sinking foundation, etc.), upgrades to windows and accessibility (i.e., ageing at home), fence and flooring renovations, and replacement of major appliances (e.g., furnaces, stove, dishwasher, washing machine, etc.). Minor repairs needed included lighting upgrades, installation of water filtration systems, and faucets replacements.

Furthermore, participants spoke about the emotional toll of being unable to afford or access necessary repairs. Many faced financial barriers, unclear eligibility criteria for support programs, or uncooperative landlords who refused to carry out repairs.

*“I have plumbing problems. I have foundation problems. I have problems all over… I’m not the only one living in this kind of place. It is an illegal duplex. So at any time*, *If the town decides that this guy [landlord] can’t do this anymore*,* I’m out.”* (FG#6)

Finding reliable contractors and obtaining supplies at a reasonable price and timeline was another major concern. This was especially difficult in certain regions where MNO citizens lived, particularly in remote areas with limited contractors.

“*I know all too well. I put my feelers out [for]… anyone that can do this work and… I get maybe two responses and neither… are good options.*” (FG#4)

##### Suitability

Participants mainly expressed a need for more living space. Some were temporarily experiencing overcrowding after moving back in with family or having family members move in due to the COVID-19 pandemic. Others shared that their homes had become too small for their growing families, but they were unable to move due to the high market price for larger dwellings. A few participants also noted that they would only consider having children if they had a bigger living space. Limited living space contributed to a lack of privacy and could contribute to tensions between occupants.

*“I lived in the city of Peterborough*,* in a 2-bedroom apartment*,* with… my husband and our 3 children. So it was small. Then we bought this house in 2018*,* and it was a 2-bedroom house*,* but we… change the basement to make a third bedroom. And… had a fourth baby… It is overcrowded*,* I would say*,* because we have 3 bedrooms with 6 people*,* and one of the bedrooms is small. So our toddler now is 3*,* and… she’s not big enough to be in a bunk bed. We have 2 sons and 2 daughters*,* so the boys have a room in the basement*,* they have a bunk bed. and my daughter*,* who’s 12*,* has her own room*,* but the 3 year old can’t sleep in there because it’s not safe to be in a bunk bed*,* so our 3 year old sleeps in a toddler bed in our room…So not ideal.”* (FG#7)

*“My home is a smaller house so if I ever wanted to have children*,* we would probably need a bigger space. It is enough for two people and two dogs but not more.” (FG#6)*

Overcrowding and a lack of living space caused feelings of frustration, helplessness, and a lack of control over housing conditions.

##### Pests and other environmental concerns

Experiences with mold and mildew, and pests (e.g., rats, bed bugs, cockroaches) were also shared. Mold and mildew were a major concern for those participants. The effects on mental and physical health are discussed in Sect. 3.5. One participant described the challenges of living in an old house prone to mold and mildew, and this underscores how inadequate housing conditions, coupled with limited resources, directly impacts participants’ ability to maintain a healthy living environment.

*“I lived in a pre 1890 construction house*,* too close to Lake Muskoka*,* and our mold and mildew was really bad*,* and it’s expensive to fix. To ventilate an old house is a lot of work.”* (FG#1)

#### Renting

This section explores the impacts of landlord-tenant relationships and the barriers to accessing quality housing as well as legal services and support programs for housing.

##### Landlord-tenant relationships

When exploring landlord-tenant relationships, imbalanced power dynamics often disadvantaged tenants. When looking for rental housing, instances of discrimination against pet ownership or familial status from landlords made the search for quality housing more limited.

*“I didn’t want to be in the terrible housing. But I was limited to my housing because I had pets*,* and people have this assumption that when you have two fifty-pound dogs that you’re going to wreck the place.” (FG#5)*

These experiences influenced not only their housing situation but also their overall wellbeing, safety, security, and sense of belonging.

Participants also shared that along with unfair treatment, lack of maintenance in rental properties and disputes over lease agreements are common occurrences and heightened stress. Inadequate responses from landlords to serious problems such as unsafe water, leaks, and mold directly impacted participant’s health and quality of life:

*“When I was living in the county. The water was unsafe*,* it had high E. coli levels. Landlord didn’t want to fix it. So there was just lots of issues… landlords not wanting to repair issues*,* leaks or mold… just a lot of issues renting.” (FG#2)*

Moreover, renters paying below market rent felt vulnerable, worried their landlords might evict them in order to raise rent:

*“I am well below market value rent right now. And I know that my current landlord*,* if I were to move out*,* he’d love it because he could check the rent up.”* (FG#2).

These experiences illustrate how laws and regulations for rental housing lack accountability and can often put the tenant in a situation where their mental, emotional, and physical health is impacted negatively by the quality of the housing they are living in.

##### Long waitlists for housing law programs/assistance

Housing programs and legal assistance for housing were described as difficult to access. For instance, one participant shared their observation of the increasing amount of time it takes to get housing through housing assistance programs:

*“Twenty years ago*,* the waitlist was three to five years. Now the wait list is anywhere from eight to fifteen. And so now*,* thinking about somebody who requires a home today. The hope isn’t there.”* (FG#3)

In Ontario, it can take years to access housing through municipal housing programs. This is a frustrating and discouraging experience that impacts mental wellbeing and creates a sense of hopelessness. Similar frustrations were expressed with the housing programs provided by the MNO. Participants provided feedback on how the MNO can improve its existing housing programs and resources. Some suggestions included expanding existing programs for housing; developing new program/resources (e.g., new programs for seniors on a fixed income; offering expanded services for snow shoveling for seniors or citizens who are unable to do it themselves, etc.); creating MNO-owned housing (e.g., community housing, rent-to-own housing, etc.); reducing program barriers (e.g., simplified application process, inclusion of citizens who are not tech-savvy, etc.); re-evaluating the eligibility criteria to reflect the current economy and on a case-by-case basis; and advertising more housing programs and supports that MNO citizens are eligible for (e.g., federal, provincial, MNO, etc.).

Others highlighted the uncertainty and anxiety that comes with not knowing if they will be accepted into the housing program or when they will be notified about their waitlist status. While programs like the MNO Emergency Repair program was seen as a potential source of assistance, the slow and unclear application process led to additional stress, especially for homeowners living with serious repair needs and no immediate solutions:

*“My husband and I*,* we own this house*,* but we cannot afford to fix any of the major repairs ourselves without assistance*,* which is why we applied to the MNO Emergency housing program. But*,* yet again we don’t know if we will even be approved for it. It’s 4 weeks wait*,* and now we’re in that anxious period… But if we don’t get it*,* how are we going to fix our home? And will this section of my house collapse? We don’t know. And so that’s a huge stress every day.”* (FG#6)

#### Affordability of shelter costs

This section looks at shelter costs, specifically overpriced rent and mortgages, housing shortages and insecurity, and the cost of housing repairs. Participants, specifically seniors, reflected on their observations of the housing market and their concerns for the affordability of housing for themselves in the future when downsizing and for the younger generations when buying their first homes. These discussions included experiences of housing insecurity and how close participants were to experiencing housing insecurity.

##### First-time home buying and high rent costs

A consistent themed across groups was how unaffordable shelter costs have become. Shelter costs are the monthly dwelling-related expenses paid by households, such as mortgage or rent, property taxes and condominium fees (for owner-occupied dwellings), and utilities (e.g., electricity, heat, water) [28]. Some participants reported allocating at least 50% of their income towards shelter costs, which is higher than the 30% threshold used to defined affordable housing. This financial strain caused by rising shelter costs have forced individuals to accept substandard housing. The financial stress caused by rising shelter costs that force one to live in poor quality housing was described as causing negative impacts on mental and emotional wellbeing.

*“It’s crazy. How we survive is we put all our money on rent*,* and then we hope like hell that we can feed everybody else. How I do it*,* is I put all my money on rent. I pay for all the cat stuff*,* and I worry about me last*,* because my rent is 1*,*500 bucks a month*,* and my monthly income is $1*,*100. Do the math! So*,* I’m always behind on rent." (FG#6)*

Participants also noted a substantial increase in market rent after the COVID-19 pandemic, where housing conditions were perceived as not worth the shelter costs. Despite believing the shelter was not worth the price, participants felt they had no other choices due to housing shortages in certain regions. Older participants reflected on how drastically the housing market had changed, with many sharing that they would not be able to afford their own homes if buying today. This raised additional worries about future downsizing and the ability of the younger generations, including their children, to buy their own homes.

*“You kind of get stuck where you’re at*,* and we know that we can’t leave while my husband and I would like to move to a different place. He’s retired and I’m working. I plan to work for a few more years*,* but once we’re no longer working… we won’t be able to afford accommodations elsewhere where the rent could be so much higher.… we’re only receiving CPP [Canadian Pension Plan] and OAS [Old Age Security– a monthly pension provided by the Canadian government to seniors aged 65+].” (FG#4)*

Additionally, some participants recounted experiences of homelessness or unstable housing throughout their lives. The sense of housing insecurity was further compounded by the high cost of housing, leading participants to worry about potential homelessness in the event of job loss.

*“I have been stressed by my housing situation. I lived 4 years terrified every single day that I would lose my house. As a single parent*,* this was traumatic for me. I became sick and disabled. lack of income to live. unable to repair and maintain [my]home.”* (FG#6)

For younger participants, the dream of homeownership felt increasingly out of reach, creating a sense of guilt, uncertainty, and hopelessness about their financial futures:

*“I’m [mid-twenties]… and I don’t know when I’m going to be able to afford a house*,* and even sometimes I feel guilty living in [large city in Southern Ontario]*,* just because I know I should be living at my parents and saving to one day be able to buy a place to live."* (FG#3)

These narratives highlight how unaffordable housing directly undermines financial security and contributes to chronic stress, particularly among low-income, young, and seniors of the MNO citizens.

##### Cost of housing repairs and aging at home

All groups discussed the challenge of affording necessary home repairs. Many participants shared current or past experiences where they were unable to afford necessary home repairs, ranging from structural issues to minor repairs, and even essential appliance replacements such as furnaces and stoves. The inability to afford repairs stemmed from various factors, such as seniors being on a fixed income, unexpected repairs, or the cost exceeding their initial budget.

*“Being retired and on a fixed income. For example*,* we needed to upgrade our insulation on the outside*,* so we’ve been gradually doing each side of the house*,* but we have to budget for that. We have to plan a year in advance that we need x amount of dollars*,* and we do the work ourselves. So*,* it’s just*,* well*,* that the cost of materials is going through the roof. We still have one more side of our house to do*,* but it does make a difference as far as heating.”* (FG#2)

Aging-at-home was highlighted as a pressing priority by our participants, since more aging MNO citizens will need to age-at-home as the median wait times for long term care homes in Ontario is 201 days in 2023 [29]. Ensuring MNO citizens’ homes are safe to age in, in which one of many barriers is cost, will become a pressing priority according to participants.

*“There’s going to be a lot of people in that certain demographic who are becoming seniors… my parents included. There’s going to be a lot of people looking for housing*,* particularly… retirement homes and medical care homes… there’s going to be really long waiting list to get into these places. So any kind of support around that… would be useful.”* (FG#3)

The financial strain of not being able to afford repairs has caused significant stress to participants. Anxiety around not being able to afford repairs made some participants avoid investigating suspected unsafe housing conditions. In addition, the rising material costs and supply chain delays that arose during the COVID-19 pandemic impacted the essential renovation plans of many participants. This further increased anxiety for participants trying to maintain safe housing conditions, especially those with children:

*“Covid really affected the expenses of everything in the way of living*,* and I don’t know. Is it Covid? Or was the global economy going in this way? But it’s really prohibited some of the things from moving forward. As I said*,* we have renovations planned*,* but it’s really put a stop on a lot of them. You know*,* my deck at this point*,* it’s at the point where it’s not safe. I don’t want my child playing on it*,* so I’m afraid.”* (FG#5)

##### Housing shortages

This theme was prevalent across all regions, as participants shared local stories indicating the limited housing options available. Limited availability forced many community members, including some participants themselves to accept substandard housing conditions, including ones with pest infestations, in disrepair, and overcrowding. Despite these conditions, participants expressed a sense of resignation, feeling they had little choice due to the broader housing crisis.

*“People have gotten crazy with the pricing of [housing]… with the buying and selling market along with the rents prior to the pandemic. It was bad. I was constantly having clients saying*,* I can’t afford this place. This place is bad. There’s bugs.… Now they’re like we’re just happy to have a place to live*,* but we’re dealing with the bugs*,*… we’re putting up with it*,* and they’re swallowing a lot more than they should*,* and they’re putting up with a lot more than they should*,* because they have a place to live.”* (FG#5)

In addition, participants described a troubling cycle where the housing supply has failed to meet the demand, thereby driving up housing cost. With limited housing, individuals were forced to stay in their current homes, because no better alternatives existed. As a result, starter homes were being renovated and removed from the entry-level market, making it even harder for first-time buyers to access affordable housing.

*“Kenora is exactly the same. it’s a bad situation… there is a lack of housing everywhere. And so*,* part of the problem is that people who have gone into a starter home*,* they have nowhere to climb up to*,* and so they’re renovating the starter homes which makes them unaffordable because they’re no longer a starter home. So*,* you just have this lack of starter home… nobody can really afford.”* (FG#2)

This shortage has also affected seniors who want to downsize but cannot find suitable housing in their communities, leading to further strain on the housing supply.

#### Impact on health

Three aspects of wholistic health were emphasized during these conversations: mental health, emotional health, and physical health. In some cases, the stress of a participant’s housing situation brought on physical illness and vice versa; housing conditions causing ill health impacted mental and emotional health negatively.

##### Mental and emotional health

It is clear from these conversations that the impact of rising cost of living on the housing market leading to overpriced mortgages and rentals, a lack of income to repair and maintain homes, and housing shortages and overpriced rents forcing participants, in some cases, to accept poor quality or inadequate housing negatively impacted the mental and emotional health of our participants. Housing is a fundamental social determinant of health and the precarity of housing in Ontario has impacted the mental and emotional wellbeing of MNO citizens, as well as the general population in Ontario, due to the unaffordability of housing and shelter costs. This has created a feeling of being stuck for many and created anxiety about current and future housing situations. For example, homeowners, are worried they will not be able to afford their mortgages when they are re-assessed, senior MNO citizens are worried they will not be able to afford to downsize, and young MNO citizens are worried they will never be able to afford a home. Owning a home creates a more stable financial future and some young MNO citizens feel they will not be able to achieve this:

“*I just know that I’m never really going to be able to afford a home. I’m [early thirties] and when you can’t* [afford a home], [you]…*get trapped into poverty.*” (FG#2).

Social pressure from social media, peers, friends, and family also played a role. Some felt judged for living in older homes or smaller spaces.

“*I sometimes feel judged because we live in an older house…my children… ask if we could move to a newer subdivision* [and] *some acquaintances (not friends) say that I live in a small house.*” (FG#7).

Comparisons between homes seen on social media platforms like Instagram and Pinterest, which are often unattainable, made participants self-conscious of their homes.

Stress and anxiety were common among homeowners who couldn’t afford repairs. Living with unresolved issues, such as mold and pests, caused constant worry. Participants feared the cost and consequences of fixing hidden problems.

“*Right now in our one of our bathrooms…there’s black mold under the floor. I can see it… but tearing it up scares me thinking*,* oh*,* what is that going to lead to?”* (FG#7)

Others shared trauma from unsafe neighborhoods, including break-ins that left them feeling scared in their own homes.

“*My apartment was broken into… not just one*,* we had 3 intruders* [break into] *our home…I was absolutely terrif*[ied]*…the police could not do anything… afterwards*, [I] *felt uncomfortable in* [our] *home…* [I] *wonder*[ed] *am I safe? Is my family safe?…* [is] *this* [going to] *happen again*?” (FG#6)

Although most participants described negative mental health impacts, participants shared how secure, affordable housing in a safe areas made a big difference in their lives.

“*Finally moving into a living situation that met my needs changed everything for me…I have a chronic illness* [and] *I have* [not] *been able to work or go back to school for over a decade*,* and finally… two years ago*,* my parents helped me get into a* [housing*] situation that works…and that completely changed my health*,* because I finally had this good balance… I had…privacy and… safety… my health*,* went through the roof*. *I immediately started to improve physically*,* emotionally*,* spiritually*,* mentally*,* and back in school. Now* [I am] *able to work… having a good… housing situation can completely change the trajectory of somebody’s life.*” (FG#2)

Access to nature, being close to their kin, having sufficient amenities nearby, and having privacy helped improve their mental, emotional, and physical wellbeing.

*“The one reason why despite the condition of the house I own right now*,* I am feeling I am in a better place*,* because I like the city I reside in now*,* which is Hamilton. I like that my house is right on the escarpment*,* so I just have the big mountain behind me*,* and I have a lot of parks around. I feel like I can breathe. When we’re in Toronto*,* I felt like I could not breathe at all*,* and I feel like that really contributes to a healthy home. It’s based on the person’s experience and the personality. But for me*,* close in nature meant a lot.” (FG#6)*

##### Physical health

Participants across all groups shared experiences of various physical illnesses, such as allergies, respiratory issues, persistent coughs, and sleeping problems, linked to their housing conditions, including mold, dampness, cockroach infestation, and environmental pollution.

For example, a participant who lived in a moldy, damp, small, poorly ventilated units experienced persistent coughing and lung problems:

“*I’ve had to go to my doctor*… [Because of] *how damp it is* [in the basement], *and with the mold… it’s affected my lungs… I didn’t even realize how much I would cough when I lived in the basement* [apartment]… *due to the mold issues… it* [was] *small* [and] *damp… it* [was] *what I could afford.*” (FG#4)

Participants also highlighted exposure to toxic materials such as asbestos, and the long-term impacts of environmental pollutants:

“[My] city…in terms of health [is] *not… the best place to live…we have a major problem with our steel plant…so* [the city is] *known* [as] *being the cancer belt. It* [has*]… been overlooked…I grew up around the steel plant*,* and we would see film on cars in the morning* [in the] *…certain areas of town* [where] *people…would be prone to cancer…*” (FG#5)

Overcrowded housing not only increased stress but also disrupted sleep, contributing to sleep disorders:

“*When I was at [university]*,* the housing around the university was… overcrowded… [*with] *seven girls living in a small house…it* [was] noisy and… that…really affected [me]*… I found out that I have a sleep disorder.”* (FG#2).

#### Infrastructure and connectedness

This section explores local issues such as limited infrastructure and safety in some communities as well as how environmental contamination from industry and historic use of some building materials affects health.

##### Community safety

Local issues such as unsafe neighbourhoods were discussed. Some had lived with dangerous or difficult neighbours, in cities with high crime rates, or in areas where police presence was frequent, which made them feel unsafe in their own homes.

*“When I was in my first apartment*,* I was happy to have a spot*,* but when I looked back on how bad it was*,* I realized the effect it was having on me to feel so unsafe. Our door didn’t even lock properly… People regularly…came in and terrorized us and police were there a lot*,* and we witnessed a lot of unsavory things.”* (FG#4)

Some participants spoke about their experiences with discrimination based on their sexual orientation or gender identities. They expressed concerns about feeling unsafe in certain neighbourhoods and the impact on where they live.

*“Being a gay person and living in places in my lifetime. I’ve experienced the most atrocious gay bashing in my life. So it’s hard to feel safe in a lot of places*,* and that’s something that I’m always worried about. I don’t think people really know what that’s like to feel like that you always got to worry about where you’re living*,* because the people around you might not like who you are*,* just because of who you are and who comes to visit you.”* (FG#6)

##### Infrastructure and greenspace

Other issues included living far from basic amenities and services like schools, grocery stores, banks, and healthcare. This was especially difficult in Northern Ontario, where long distances and the lack of public transit meant people needed cars to get to necessary amenities and services, adding to living costs.

*“Where I live and many of the outskirts of our town there is no transportation. So*,* you have to have a car*,* and then that adds another whole expense. because there are no buses*,* and we don’t have access to Uber and those kinds of things in our community.”* (FG#5)

Environmental contamination limited green space, and weak community connections also came up as concerns. These factors shaped participants’ decisions on where to live and how livable they found their communities.

##### Family and Métis community connection

Participants expressed being close to family and community as influencing their housing decisions. Some participants lived within driving distance of family and friends, while others had to rely on neighbours or the local community for social connection and help.

*“I would feel that this would not be a great place to be struggling when you are older*,* and I’m not sure what that would look like if you didn’t have family nearby*,* which I’m not from here*,* I moved here. My closest family is more than 2 hrs away. For me*,* that’s a struggle.… there is a big sense of community locally like on my road*,* so I’m grateful for that. We do all kind of*,* like look out for each other. But not everybody has that*,* and I’m really thankful that our street does have that.”* (FG#7)

Housing designed to connect the Métis community was brought up when discussing ways to improve the MNO’s housing programs. Specifically, participants suggested the MNO build more of its own community and rent-to-own housing reserved for MNO citizens. These endeavours were explained as supporting Métis ways of living and being that goes beyond simply securing affordable safe housing for citizens.

“*I really like the idea of community…if there were to be a housing complex that focused on families and elders. We could have elders within this community bonding with these families*,* these families have more support. They’re together… it takes a village right*,* not even just to raise a child*,* but to thrive and survive. So that’s a really traditional model within our Indigeneity that we should be returning to community supporting one another*,* helping one another out.”* (FG#2)

## Discussion

This study is the first to qualitatively explore the relationship between housing conditions and the overall health of MNO citizens. It contributes to the limited literature examining the association between housing conditions and the health and wellbeing of Indigenous Peoples in Canada. Drawing upon the stories and lived experiences shared by MNO citizens, ranging from minor housing repairs to broader housing market dynamics, it is evident that housing conditions undoubtedly influence individuals’ wholistic health. This insight is crucial for the MNO, and more broadly for all Governing Members of the Métis National Council (MNC) and organizations supporting urban Indigenous Peoples with Métis clients, as housing is considered a Métis social determinant of health [3]. These results will be used to improve existing MNO housing programs and to develop new resources that will better serve MNO citizens’ housing needs, and to inform policymakers. These results also reflect the ongoing challenges across Ontario with respect to housing.

Our study provided a Métis-specific perspective on the conceptualization of healthy housing and illustrated the past and current challenges of housing and health affecting MNO citizens. Our results highlighted significant challenges with adequacy of housing for MNO citizens, with the need for major repairs in participants’ homes, causing emotional and financial strain. These findings reflect quantitative data including the 2021 Canada Census, where 10.0% of Métis lived in housing that needed major repairs, nearly twice the rate compared to the non-Indigenous population (5.7%) [15]. And in the Housing Census conducted by the MNO in 2022, it was found that 23% of MNO citizens reside in a home in need of major repairs [16]. Moreover, a mixed-method study examining housing experiences of Métis women and 2SLGBTQQIA + people found that a significant portion of respondents lived in substandard housing conditions, including landlords refusing to make repairs and unsafe living environments [30]. Being forced to live with mold or gas exposure, either due to uncooperative landlord or inability to access or afford necessary repairs, was common among the 5 Métis participants, where each of them have lived in a place that had black mold [30]. Many also reported in need of both major and minor housing repairs [30], similar housing challenges were also reflected in our findings.

Participants emphasized the unaffordability of housing in our conversations, with many allocating nearly 50% of their income towards rent and facing substandard housing conditions due to financial constraints, much higher than the less than 30% defined for affordable housing [11]. The experiences shared by MNO citizens were similar to the 2018 Canadian Housing Survey, where 55.7% of Indigenous households reported difficulty to meet their financial needs in terms of transportation, housing, food, clothing and other necessary expenses [11]. They also reflect the housing affordability crisis in Ontario more broadly [31].

Our study also highlighted the significant impact of housing conditions and unaffordability had on the mental, emotional, and physical health of MNO citizens. Most participants highlighted their housing situations were deteriorating their health and wellbeing, especially their mental, emotional, and physical health. Due to the unaffordability of housing, participants found themselves unable to leave inadequate and unsafe living conditions. The impacts of unaffordability and having to endure poor housing situations led participants to feel stressed, anxious, trapped, and judged by peers, all of which were detrimental to their mental and emotional wellbeing. These findings align with Bower et al. [32], where individuals often described feeling trapped when they did not have the financial means to improve their housing situation. Similarly, Caliyurt [33], highlighted a circular relationship between housing and mental health, where inadequate housing exacerbate stress, leading to disorders like depression and anxiety, while poor mental health renders people unable to cope with and improve their living conditions. This relationship was evident in the stories shared by participants, with one participant describing that when attending university, the stress of finding housing and affording rent increased their stress levels to the point in which they had to move back home.

As inadequate housing conditions raised the stress and anxiety levels of participants, having adequate housing conditions improved their mental health. Participants shared feeling proud and in control of their wellbeing when living in safe neighbourhoods with affordable homes and access to nature. This aligns with Bond et al. [34], which highlighted the importance of both physical housing structure and neighbourhood characteristics in promoting good mental health and wellbeing. Additionally, Patino et al. [35] found that the increased proximity of greenspace near homes and peoples’ self-reported happiness were positively correlated with each other. Our study further corroborates these findings, with participants expressing increased wellbeing after moving to a city where they were surrounded by nature. Access to the land and nature is of particular importance of Métis people as land is intrinsically tied to their culture and identity [8]. Land is also cited as a determinant of Métis health which is linked to one’s housing situation [3, 36]. As land is a foundational element of Métis culture and culture is a foundational element of Métis health, it can therefore be understood that land is vital to Métis health [8, 37]. These findings underscore the significance of both housing adequacy and neighbourhood quality in improving mental health and emphasize the importance of Métis people’s access to nature.

Participants also described a range of physical health problems as a result of inadequate housing conditions, including allergies, respiratory issues, persistent coughs, and sleep disorders. This is consistent with quantitative studies showing the prevalence of chronic obstructive pulmonary disease (COPD) is over 1.6 times higher and asthma is 1.2 times higher among Métis people in Ontario [38]. Damp and moldy housing is strongly associated with poor respiratory health, such as the development of asthma, other chronic respiratory symptoms, and recurrent headaches, fever, nausea and vomiting [2, 39]. Additionally, old, dirty carpeting and pest infestations such as cockroaches can cause allergic sensitization, trigger asthma, and other respiratory illnesses [2], which were reflected in stories shared by participants who lived in dwellings with old carpets and pest infestation. While many of these physical health problems associated with inadequate housing are not unique to Métis citizens and are reflective of broader systemic issues in Canada’s housing system [40, 41], capturing these experiences through a Métis lens remains essential to understanding how such challenges uniquely affect MNO citizens and to informing tailored supports that respond to their specific needs.

Wrap around services are a key value of the MNO’s service provision. The results of this study demonstrate how MNO citizens view the intersection of housing and health. Increased inter-departmental conversations and coordination between staff and management responsible for housing programs in the MNO and staff and management responsible for health, wellness and community wellbeing to support program development that is culturally sensitive and recognizes housing as a fundamental contributor to wholistic health has been initiated through sharing these results. The results from the study will also be used by the MNO to frame policy discussions on how to approach the needs of Métis as it related to housing, homelessness, and wholistic health. In addition, the results are being used to support advocacy for continued funding. The MNO, along with other Métis governments signed a Canada-Métis housing accord in 2018 funded by a $500 M investment over 10 years that expires in 2028. Evidence to support continued investments like the impact of inadequate and unaffordable housing on mental, emotional, and physical health will be critical.

A major strength of this study lies in being the first qualitative study to capture Métis voices regarding the impact of housing conditions on their wholistic health and wellbeing. Existing literature that explored housing and health were predominantly done with First Nations or adopted a pan-Indigenous approach. Given that the Métis are a distinct Nation with a unique culture, language, and worldview, a Métis-specific and distinction-based research approach is warranted. Métis people have often been excluded in research and by Canada in general, thus their experiences are rarely captured nor understood [42]. Furthermore, this study was led by the MNO, which ensured adherence to ethical principles of Métis research. This involvement supports Indigenous data sovereignty, affirming the rights of Indigenous Peoples to have control over numerous aspects of data, including collection, storage, ownership, access and consents, and application [43].

This study has several limitations. Most (83%) of participants were women and 86% were aged between 25 and 65, as such, the results presented in this paper may not reflect the experiences of housing health for MNO citizen men, non-binary individuals, and young people. Secondly, there was a considerable wait time between conducting the first focus group (conducted on September 6, 2022) and the subsequent sessions (conducted between November 3–8, 2022), as well as between the sixth (conducted on January 11, 2023) and seventh (February 17, 2023) focus groups. We encountered recruitment challenges where individuals who were neither MNO citizens nor residents of Canada were attempting to participate virtually, prompting us to add additional security measures to our screening survey and re-start recruitment after the first focus group. The extended intervals between focus groups reflected different housing-related developments in Ontario which may have influenced the focus of the discussions with the last two focus groups as more concerns related to the interest rate increases in December 2022 and January 2023 were brought up [44], in relation to being able to afford housing in the future. Due to our online recruitment method and use of Zoom, MNO citizens without internet access, with limited computer literacy, or with greater work and childcare demands may have been less likely to participate. This may have introduced selection bias as their experiences could differ significantly from those who were able to participate.

## Conclusion

This is the first Métis-specific study to explore the thoughts, feelings, and stories of MNO citizens regarding the impact of housing conditions on their wholistic health. Our findings revealed a multifaceted relationship between housing and health, extending beyond individual living conditions to encompass broader factors such as neighbourhood dynamics, housing market trends, and overall economic conditions. Particularly noteworthy is the significant mental and emotional health impact of housing on MNO citizens, with many reporting substantial improvements once they have moved to a better housing situation. Moreover, our study captures a wealth of valuable feedback and suggestions that will be used to inform MNO housing programs, aiming to increase Métis homeownership and improve affordable housing options, informed by a Métis understanding of health.

## Data Availability

The datasets generated and analyzed during the current study are not publicly available but are available from the corresponding author upon reasonable request, and with permission of the MNO.
